# The reproductive potential of vitrified-warmed euploid embryos declines following repeated uterine transfers

**DOI:** 10.1186/s12958-024-01192-z

**Published:** 2024-02-13

**Authors:** A. Almohammadi, F. Choucair, L. El Taha, H. Burjaq, M. Albader, A. B. Cavanillas, Johnny T. Awwad

**Affiliations:** 1 Department of Preventive Medicine and Public Health, University of Granada, Granada, 18071 11 Spain; 2 Division of Reproductive Medicine, Sidra Medicine, OPC Bldg. Level 3. Office 302. Al Luqta St. Education City North Campus. Qatar Foundation, Doha, PO BOX 26999, Qatar; 3 Department of Assisted Reproduction, Hamad Medical Corporation, Doha, 3050 Qatar; 4 Division of Reproductive Endocrinology and Infertility, Massachusetts General Hospital, Boston, MA USA

**Keywords:** Recurrent implantation failure, Preimplantation genetic testing for aneuploidy, Euploid blastocyst rate, Sustained implantation, Live birth rate

## Abstract

**Background:**

Recurrent implantation failure (RIF) represents a vague clinical condition with an unclear diagnostic challenge that lacks solid scientific underpinning. Although euploid embryos have demonstrated consistent implantation capabilities across various age groups, a unanimous agreement regarding the advantages of preimplantation genetic testing for aneuploidy (PGT-A) in managing RIF is absent. The ongoing discussion about whether chromosomal aneuploidy in embryos significantly contributes to recurrent implantation failure remains unsettled. Despite active discussions in recent times, a universally accepted characterization of recurrent implantation failure remains elusive. We aimed in this study to measure the reproductive performance of vitrified-warmed euploid embryos transferred to the uterus in successive cycles.

**Methods:**

This observational cohort study included women (*n* = 387) with an anatomically normal uterus who underwent oocyte retrieval for PGT-A treatment with at least one biopsied blastocyst, between January 2017 and December 2021 at a university-affiliated public fertility center. The procedures involved in this study included ICSI, blastocyst culture, trophectoderm biopsy and comprehensive 24-chromosome analysis of preimplantation embryos using Next Generation Sequencing (NGS). Women, who failed a vitrified-warmed euploid embryo transfer, had successive blastocyst transfer cycles (FET) for a total of three using remaining cryopreserved euploid blastocysts from the same oocyte retrieval cycle. The primary endpoints were sustained implantation rate (SIR) and live birth rate (LBR) per vitrified-warmed single euploid embryo. The secondary endpoints were mean euploidy rate (m-ER) per cohort of biopsied blastocysts from each patient, as well as pregnancy and miscarriage rates.

**Results:**

The mean age of the patient population was 33.4 years (95% CI 32.8–33.9). A total of 1,641 embryos derived from the first oocyte retrieval cycle were biopsied and screened. We found no associations between the m-ER and the number of previous failed IVF cycles among different ranges of maternal age at oocyte retrieval (*P* = 0.45). Pairwise comparisons showed a significant decrease in the sustained implantation rate (44.7% vs. 30%; *P* = 0.01) and the livebirth rate per single euploid blastocyst (37.1% vs. 25%; *P* = 0.02) between the 1st and 3rd FET. The cumulative SIR and LBR after up to three successive single embryo transfers were 77.1% and 68.8%, respectively. We found that the live birth rate of the first vitrified-warmed euploid blastocyst transferred decreased significantly with the increasing number of previously failed IVF attempts by categories (45.3% vs. 35.8% vs. 27.6%; *P* = 0.04). A comparable decrease in sustained implantation rate was also observed but did not reach statistical significance (50% vs. 44.2 vs. 37.9%; *P* = NS). Using a logistic regression model, we confirmed the presence of a negative association between the number of previous IVF failed attempts and the live birth rate per embryo transfer cycle (OR = 0.76; 95% CI 0.62–0.94; *P* = 0.01).

**Conclusions:**

These findings are vital for enhancing patient counseling and refining management strategies for individuals facing recurrent implantation failure. By tailoring interventions based on age and ovarian reserve, healthcare professionals can offer more personalized guidance, potentially improving the overall success rates and patient experiences in fertility treatments.

**Trial registration number:**

N/A.

## Background

Recurrent implantation failure (RIF) is an ill-defined clinical entity and poorly understood diagnostic dilemma that lacks sound scientific basis [1, 2]. It is characterized by the failure to achieve pregnancy defined by negative human chorionic gonadotropin levels despite repeated embryo transfers (ETs). Although common RIF definitions agree on the concept of recurrence, the number of failed attempts is not well established and proposed numerical values lack rational scientific foundation. Beyond the number of previously unsuccessful in vitro fertilization (IVF) cycles, some definitions take into consideration the number, quality and developmental stage of transferred embryos [3–5]. Other characteristics important to the process of implantation are nonetheless not considered, such as endometrial receptivity, sperm quality and laboratory factors [6]. With increasing utilization of pre-implantation genetic testing for aneuploidy (PGT-A), it is now clear that embryo euploidy rate is a function of maternal age, and that chromosomally normal embryos are more likely to implant and develop into a sustained pregnancy [7]. Until very recently, RIF definitions neither stratified for maternal age nor excluded aneuploid embryos, which caused an overdiagnosis of the condition, mostly in women with advanced age and low ovarian reserve [3–5].

Understandably, humans by nature are slow breeders as they express a maximum monthly fecundability of 20–25% during the first few months of exposure [8]. The limited implantation potential of human embryos has significant implications on the success of an IVF cycle. Despite advances in the field of assisted reproduction, it is estimated that 35% of euploid embryos transferred to an anatomically normal uterus fail to implant causing many patients to experience the bitterness of failure after multiple IVF cycles and to worry about the uncertainty of the prospects of future pregnancy [2, 9]. This has rendered the timing of an investigative work-up difficult to determine, which has increased population heterogeneity and compromised the interpretability of the medical literature, hampering future research in the field of implantation failure [10]. Even more challenging is the non-specific nature of RIF definitions which makes it hard to expose genuine etiologies, leading to an alarming trend of overdiagnosis and overtreatment [9]. Not unexpectedly, many RIF investigations are concluded with unmet expectations [3] bringing more empiric treatments onboard fueled by anxious couples and frustrated care providers. Despite plausible biologic rationale, many treatments have little evidence to support their clinical effectiveness subjecting patients to the potential risks of these unwarranted interventions [11].

The complex process of embryo implantation includes several interactions and pathways that involve the embryo, endometrium, and immune system [12]. It is believed that at least 50–65% of the implantation potential of an embryo is accounted for by its euploid status [13]. In the remaining 35–50%, implantation failure occurs in the absence of a clear etiology. Recurrent implantation failure remains therefore an ill-defined clinical entity largely idiopathic in nature [1, 2]. While euploid embryos have been shown to maintain their implantation potential across different age strata [14, 15], there is no universal consensus on the benefits of PGT-A in RIF management [16], namely when a significant population of women with low follicular response fails the eligibility criteria for pre-implantation genetic screening [7, 13, 17, 18]. While the debate over whether embryonic chromosomal aneuploidy is a major contributor to recurrent implantation failure is still ongoing, PGT-A is not an established strategy to address the condition [13, 17, 18]. It has been suggested that the use of PGT-A, while not addressing the underlying pathophysiology, could offer a pragmatic approach that favors the selection of embryos based on a predominant and well-established determinant of implantation success.

Despite heated debates in recent years, a universal definition of recurrent implantation failure is far from being established. The primary objective of this study was to address this existing data gap and generate hypotheses by (a) measuring the reproductive performance of vitrified-warmed euploid blastocysts collected from the same oocyte retrieval cycle across consecutive embryo transfers, and (b) determining associations between the clinical outcomes of the first euploid embryo transfer and past reproductive history, namely previous IVF failures. To accomplish this aim, we retrospectively studied women undergoing oocyte retrievals for PGT-A in a university-affiliated public fertility center between January 2017 and December 2021. Generated data is expected to improve our understanding of RIF, guide future clinical research, and counsel patients with past repeated failures about the benefits of PGT-A in improving their reproductive outcomes.

## Methods

### Study population

We conducted an observational study of women who underwent oocyte retrieval for PGT-A treatment cycle with at least one biopsied blastocyst, between January 2017 and December 2021 at a university-affiliated public hospital (Department of Assisted Reproduction at the Woman Wellness Research Center) in Qatar. All patients in the study population were between the age of 18 and 45 years, had a body mass index (BMI) of > 18 kg/m2 and < 40 kg/m2, and a morphologically normal uterus on saline sonography and/or hysteroscopy.

Women who failed a cryo-warmed euploid embryo transfer had successive vitrified-warmed euploid blastocyst transfer cycles (FET) for a total of three using remaining cryopreserved euploid blastocysts from the same oocyte retrieval cycle. At each autologous FET, the embryo with the best morphology as determined by Gardner scoring system was prioritized for transfer. Embryos were considered good-quality when at least the blastocoel filled the entire embryo (grade 3), the inner cell mass was loosely packed with several cells (grade B), and the trophectoderm had fewer cells in a loose layer (grade B). Good-quality embryos hence included 3–6AA/AB/BA/BB, while poor-quality embryos did not meet these criteria. The KIDS score was utilized only to discriminate between embryos of similar morphological grading. All embryos underwent trophectoderm (TE) biopsy and PGT-A at the blastocyst stage using next generation sequencing-based platform from Igenomix (Barcelona, Spain), followed by vitrification. Endometrial preparation was performed using oral estradiol and intravaginal progesterone supplementation. The transfer of a euploid autologous blastocyst was performed on the sixth day of progesterone initiation in the presence of an endometrial thickness of ≥ 7 mm.

### Ethical approval

Institutional review board (IRB) approval was obtained from Hamad Medical Corporation Hospitals for this study (MRC-01-22-181).

### Patient treatment

#### Ovarian stimulation

Controlled ovarian stimulation (COS) was performed using recombinant follicle stimulating hormone (Gonal-F®; recFSH, Merck Serono, Switzerland) and/or highly purified urinary gonadotropins (Menopur®; hpHMG, Ferring, Denmark). The gonadotropin starting dose was individualized to patient characteristics and dose adjustments performed according to transvaginal ultrasound findings and estradiol serum levels. A GnRH antagonist (Cetrotide®; Cetrorelix, Merck Serono, Switzerland) was utilized for pituitary suppression, 0.25 mg subcutaneously daily, when at least one follicle attained 12–14 mm in diameter. Final oocyte maturation was triggered using a GnRH agonist (Gonapeptyl®; triptorelin, Ferring, Denmark). Transvaginal oocyte retrieval was performed 36 h after GnRH agonist 0.3 mg subcutaneous administration. Assisted fertilization was performed using intracytoplasmic sperm injection (ICSI). Normally fertilized zygotes were cultured in extended culture medium in a time lapse incubator (Embryoscope®; Vitrolife, Sweden). Blastocysts were considered suitable for TE biopsy and vitrification based on a morphokinetic assessment that combines the criteria of Gardner and Schoolcraft (Gardner et al., 2000) with the KID score.

#### Embryo genetic testing

Comprehensive 24-chromosome screening for aneuploidy on 6–8 trophectoderm cells at the blastocyst stage was performed at Igenomix laboratories (Barcelona, Spain) using next generation sequencing. Only euploid embryos were transferred, while mosaic and aneuploid embryos were excluded.

#### Endometrial preparation for embryo transfer

For endometrial preparation, patients received oral estradiol (Estrofem®; Novo Nordisk, Denmark) 2 mg orally twice daily for the first 4–6 days and three times daily thereafter. Endometrial thickness was monitored by transvaginal ultrasonography. Progesterone supplementation was started using vaginal progesterone (Endometrin®; Ferring, Denmark) one 100 mg vaginal insert three times daily when the endometrial thickness measured ≥ 7 mm by transvaginal ultrasonography. On the sixth day of progesterone administration, a single cryo-warmed euploid blastocyst was transferred as per policy, with some exceptions (Mean 1.27; Median 1; Range 1–2). All transfers were performed under ultrasound guidance using a soft catheter (Wallace®, Cooper surgical, USA). Hormonal supplementation was continued until 10–12 weeks of gestation. Empiric adjunct therapies were used according to providers’ discretion, but were not accounted for in the study analysis.

### Statistical analyses

Descriptive statistics [mean, proportion (%), standard deviation (SD), and 95% confidence intervals (95% CI)] were used to describe the population demographics, treatment cycle characteristics, and reproductive outcome measures (Tables 1 and 2). Normal distribution was evaluated using Kolmogorov-Smirnov analysis. The patients’ demographics were recorded on the first oocyte retrieval, irrespective of the number of consecutive embryo transfer cycles.

**Table 1:** Patient demographic and treatment cycle characteristics at oocyte retrieval

**PATIENT DEMOGRAPHICS**	
Patients undergoing oocyte retrieval for PGT-A (N)	581
Patients with at least one biopsied blastocyst (N)	387
Maternal age* [years (95% CI)]	33.4 (32.8–33.9)
≤ 29 [N (%)]	99 (25.6)
30 to 34 [N (%)]	107 (27.7)
35 to 39 [N (%)]	139 (36.0)
≥ 40 [N (%)]	41 (10.6)
Previous births [mean (95% CI)]	1.3 (1.1–1.5)
Previous miscarriages** [mean (95% CI)]	1.5 (1.1–1.8)
0 [n (%)]	222 (68.1)
1 to 2 [n (%)]	39 (12.0)
≥ 3 [n (%)]	65 (19.9)
Previous failed IVF attempts [mean (95% CI)]	2.8 (2.4–3.2)
0 [n (%)]	210 (54.3)
1 to 2 [n (%)]	96 (24.8)
≥ 3 [n (%)]	81 (20.9)
History of single gene disorder PGT-M [n (%)]	174 (45.0)
History of fetal aneuploid [n (%)]	14 (3.6)
**LABORATORY FINDINGS**	
Oocytes collected per patient [n (95% CI)]	13.4 (12.4–14.4)
Blastocysts biopsied per patient [n (95% CI)]	4.1 (3.7–4.4)
Maturation rate [% (95% CI)]	75.2 (72.4–77.9)
Fertilization rate [% (95% CI)]	76.4 (73.6–79.2)
Blastulation rate [% (95% CI)]	61.6 (56.6–66.6)
Euploid rate [% (95% CI)]	60.6 (57.1–64.2)

* Missing values: 1. ** Missing values: 61

PGT-A, Preimplantation genetic testing for aneuploidies; PGT-M, Preimplantation genetic testing for monogenic diseases

**Table 2:** Clinical outcomes of the first, second and third vitrified-warmed euploid embryo transfers

	1st FET cycle	2nd FET cycle	3rd FET cycle
Vitrified-warmed embryo transfer cycles (n)	288	63	13
Vitrified-warmed embryos transferred (n)	364	82	17
Proportion of good-quality embryos [n (%)]	**345 (94.8)** ^a,b^	75 (91.5)^a^	**13 (76.5)** ^b^
Total pregnancies per embryo transfer cycle [n (%)]	149 (53.0)	29 (49.2)	5 (38.5)
Clinical pregnancies per embryo transfer cycle [n (%)]	139 (49.4)	29 (49.2)	5 (38.5)
Livebirth rate per embryo transfer cycle [n (%)]	115 (41.1)	25 (42.4)	4 (30.8)
Miscarriages [n (%)]	32 (21.8)	4 (13.8)	1 (20.0)
Sustained implantation rate per single embryo transferred [n (%)]	**170 (46.7)** ^c,d^	33 (40.7)^c^	**5 (30.0)** ^d^
Livebirth rate per single embryo transferred [n (%)]	**135 (37.1)** ^e,f^	28 (33.9)^e^	**4 (25.0)** ^f^

*Using Fisher exact test –*^a^ 1st FET cycle vs. 2nd FET cycle (*P* = *NS*), ^b^ 1st FET cycle vs. 3rd FET cycle (*P* = 0.01),

*Using Wilcoxon signed rank test -*^c^ 1st FET cycle vs. 2nd FET cycle (*P* = *NS*), ^d^ 1st FET cycle vs. 3rd FET cycle (*P* = 0.01), ^e^ 1st FET cycle vs. 2nd FET cycle (*P* = *NS*), ^f^ 1st FET cycle vs. 3rd FET cycle (*P* = 0.02)

The primary endpoints were sustained implantation rate (SIR) and livebirth rate (LBR) per vitrified-warmed single euploid embryo. Sustained implantation was confirmed once fetal cardiac activity was detected. Livebirth was defined as a live newborn delivered after 24 weeks’ gestation. The secondary end points were mean euploidy rate (m-ER) per cohort of biopsied blastocysts from each patient, total pregnancy rate (positive hCG titer), livebirth rate per embryo transfer cycle, and miscarriage rate.

The χ2 and Fisher’s exact tests were conducted to outline putative differences among categorical variables. A general linear model was performed to evaluate associations between the m-ER as a dependent variable, and the number of past IVF failures among different ranges of maternal age. The Kruskal-Wallis test was used to compare outcomes across different IVF failure categories. Associations between clinical outcomes of the first euploid embryo transfer and selected variables were assessed using univariate and multivariate logistic regression models. Potential confounders were assessed based on statistical and biological considerations. A significance of 0.05 was set for the inclusion of variables into the multivariate model, whereas 0.1 was the cutoff for exclusion. Explored confounders included women’s age and the number of embryos transferred. Pairwise comparisons between clinical outcomes of the 1st, 2nd, and 3rd transfer were conducted using Wilcoxon paired ranked test. A *P*-value of < 0.05 was considered statistically significant. All statistical analyses were conducted with SPSS (Release 27, IBM, USA).

## Results

### Patient demographic and treatment cycle characteristics

**Fig. 1:**
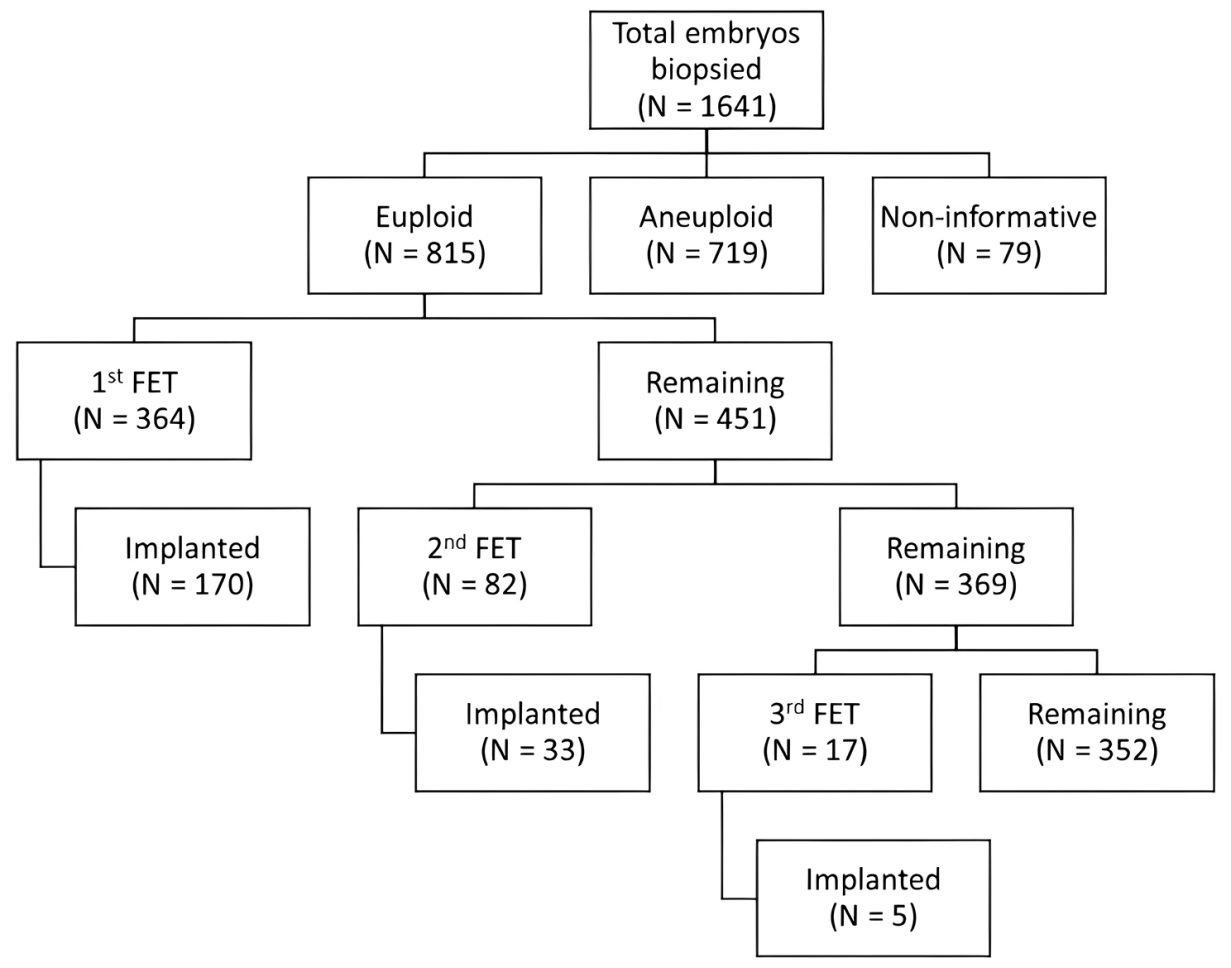
Flow diagram representing the implantation of vitrified warmed euploid blastocysts after up to three consecutive uterine transfers

A total of 581 couples underwent oocyte retrieval for PGT-A during the study period, 387 of whom had at least one blastocyst available for trophectoderm biopsy. A total of 1,641 embryos derived from the first oocyte retrieval cycle were biopsied: 815 were euploid, 719 were aneuploid or mosaic, and 79 were uninformative (Fig. 1).

The mean age of the patient population was 33.4 years (95% CI 32.8–33.9). Patient demographics (PGT-A indications, previous births, previous miscarriages, previous failed IVF attempts) are illustrated in Table 1. We defined four categories of maternal age at oocyte retrieval (≤ 29/30–34/35–39/≥40) and three for the number of previously failed IVF cycles (0/1–2/≥3). During the first oocyte retrieval, participants had an average of 13.4 oocytes (95% CI 12.4–14.4) collected and 4.1 blastocysts (95% CI 12.4–14.4) biopsied. The maturation, fertilization, and blastulation rates were 75.2% (95% CI 72.4–77.9), 76.4% (95% CI 73.6–79.2), and 61.6% (95% CI 56.6–66.6), respectively.

**Fig. 2:**
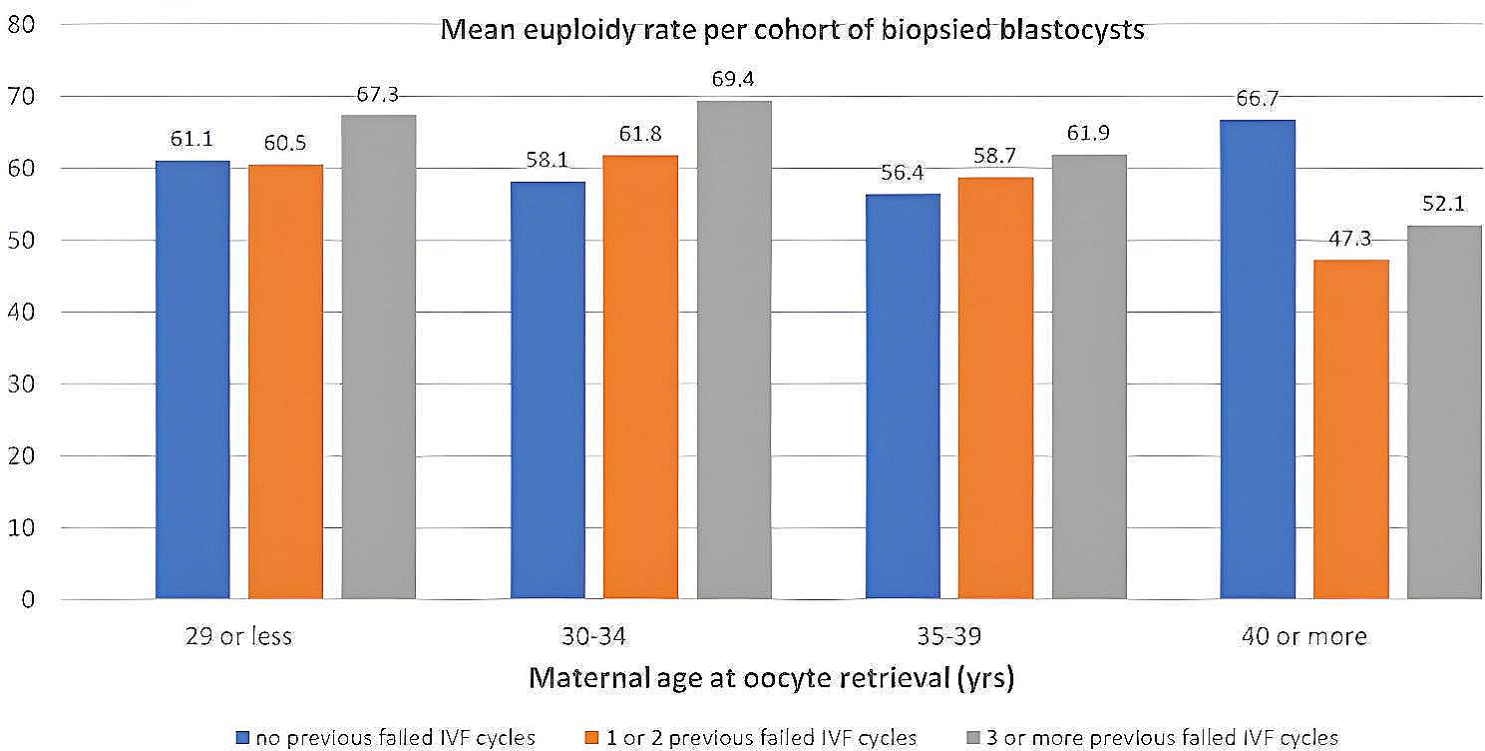
Mean euploidy rate per cohort of biopsied blastocysts from each patient according to the number of previously failed IVF cycles (0/1–2/≥3) and among different ranges of maternal age at oocyte retrieval ≤ 29/30–34/35–39/≥40). General linear regression analysis found no associations between m-ER and the number of previous failed IVF cycles among different ranges of maternal age at oocyte retrieval (*P* = 0.45)

The mean euploidy rate per cohort of biopsied blastocysts (m-ER) was 60.6 (95% CI 57.1–64.2). A linear regression model was used to test if maternal age significantly predicted m-ER. We found that maternal age significantly predicted m-ER (R^2^ = 0.018, F (1,378) = 7.917, ß = -0.143, *P* = 0.005). We also conducted a General Linear Model analysis to study the effects of the number of previously failed IVF attempts on m-ER with maternal age as covariate (data shown in Fig. 2). We found no evidence that the number of previous failed IVF cycles was a significant predictor of m-ER among different ranges of maternal age at oocyte retrieval (R^2^ = 0.038, F [1, 2] = 0.792, *P* = 0.45). The data is consistent with the null hypothesis.

### Primary clinical outcomes

**Fig. 3:**
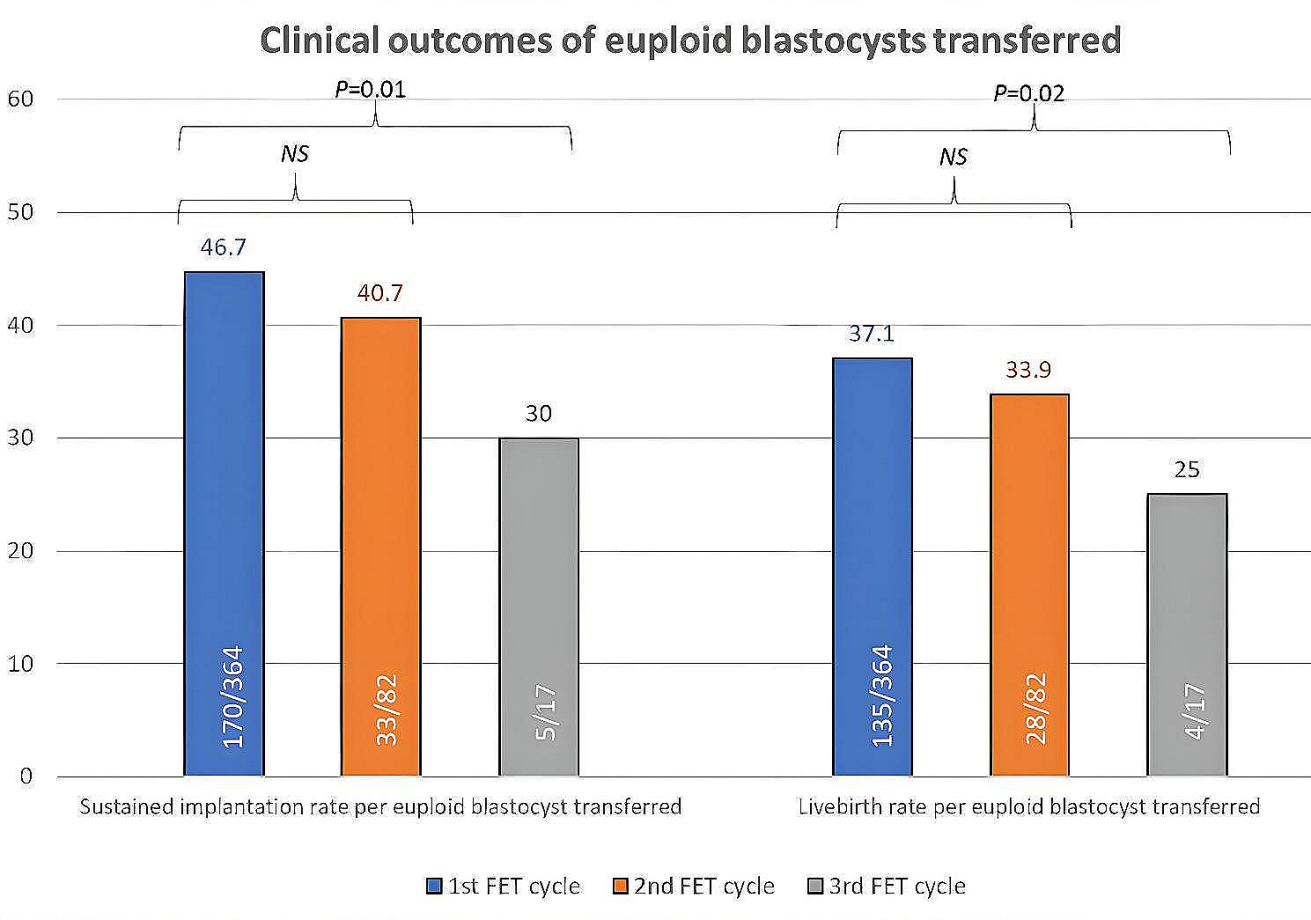
Clinical outcomes of euploid blastocyst transferred during the first, second, and third cycles. Using Wilkinson paired ranked test, pairwise comparisons between the 1st and 3rd FET showed a significant decrease in the sustained implantation rate (46.7% vs. 30%; *P* = 0.01) and the livebirth rate per single euploid blastocyst (37.1% vs. 25%; *P* = 0.02)

Using Wilcoxon signed rank test, pairwise comparisons showed a significant decrease in the sustained implantation rate (44.7% vs. 30%; *P* = 0.01) and the livebirth rate per single euploid blastocyst (37.1% vs. 25%; *P* = 0.02) between the 1st and 3rd FET (Table 2; Fig. 3). Using Kaplan-Meier survival analysis, the cumulative SIR and LBR after up to three successive single embryo transfers, assuming all euploid blastocysts were transferred, were 77.1% and 68.8%, respectively. Stratification by embryo grading showed that a significantly higher proportion of good-quality embryos were replaced in the 1st FET compared to the 3rd FET (94.8% vs. 76.5%; *P* = 0.01).

**Fig. 4:**
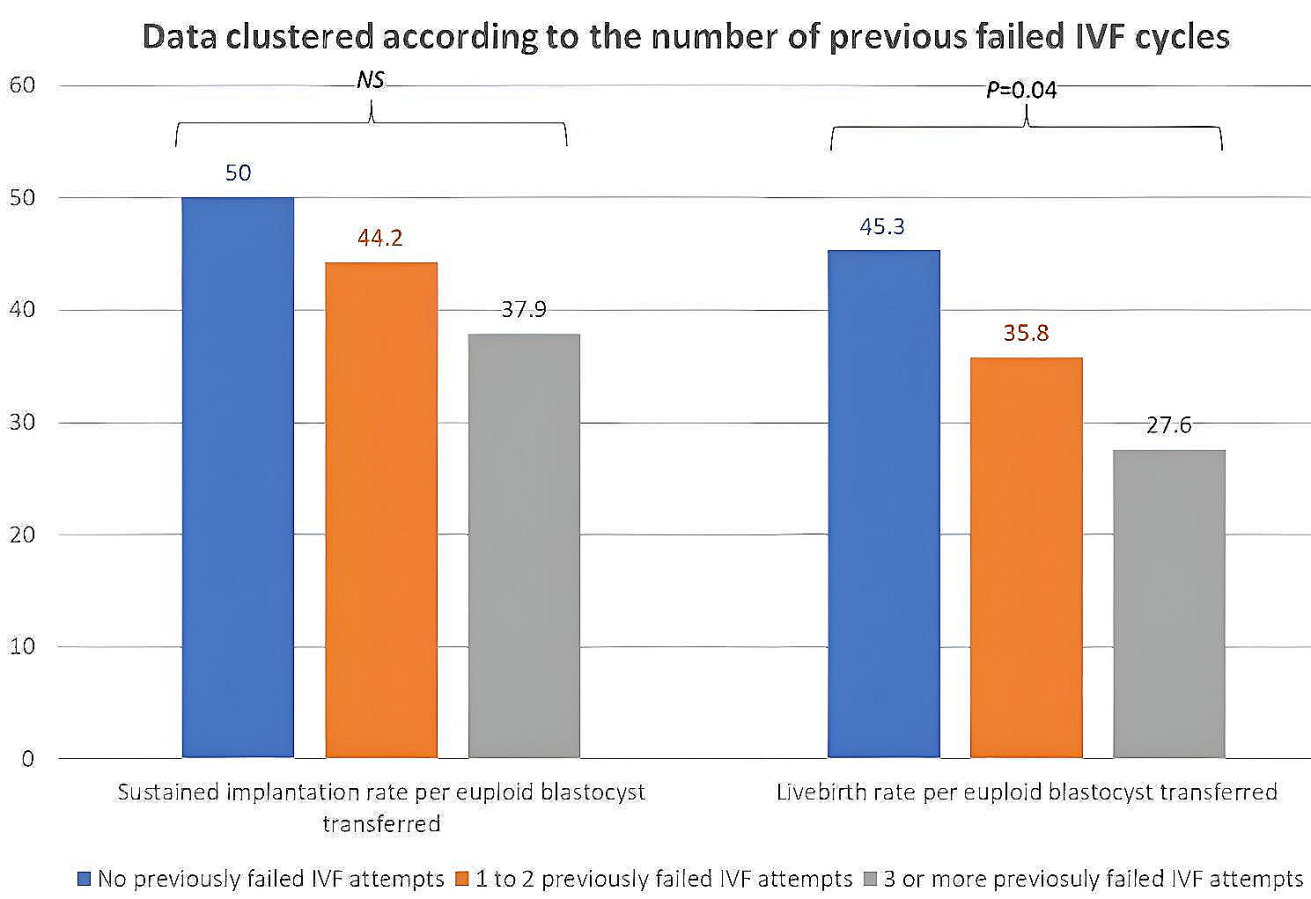
Clinical outcomes of the first vitrified warmed euploid blastocyst transferred: Data clustered according to number of previous failed IVF cycles. Using the Kruskal Wallis test, the livebirth rate per single euploid embryo was found to decrease significantly with an increasing number of previously failed IVF attempts (45.5% vs. 35.8% vs. 27.6%; *P* = 0.04)

We used the Kruskal-Wallis test to study the clinical outcomes of the first vitrified-warmed euploid blastocyst transfer according to a history of past implantation failures (Fig. 4). We found that the livebirth rate per single euploid embryo decreased significantly with the increasing number of previously failed IVF attempts by categories (45.5% vs. 35.8% vs. 27.6%; *P* = 0.04). A comparable decrease in sustained implantation rate was also observed but did not reach statistical significance (50% vs. 44.25 vs. 37.9%; *P* = NS).

### Secondary clinical outcomes

As shown in Table 2, a total of 288 women underwent the 1st FET. Of those who failed to achieve implantation (*n* = 140), 63 underwent a 2nd FET. Of those who failed to achieve implantation (*n* = 34), 13 patients underwent a 3rd FET. The total pregnancy rates after the 1st, 2^nd,^ and 3rd FET were 53.0%, 49.2%, and 38.5%, respectively.

Using a logistic regression model, we analyzed the clinical outcomes of euploid embryos after the 1st FET (Table 3). We found a negative association between the number of previous IVF failed attempts and the livebirth rate per embryo transfer cycle (OR = 0.76; 95% CI 0.62–0.94; *P* = 0.01). No associations were found between the number of previous IVF failed attempts and clinical pregnancy rate (OR = 0.59; 95% CI 0.32–1.07; *P* = 0.08) or the miscarriage rate (OR = 2.5; 95% CI 0.96–6.62; *P* = 0.06). Patients with a history of ≥ 3 previous miscarriages accounted for 19.9% of the patient population (Table 1). When a logistic regression model was used, we detected a positive association between the number of previous pregnancy losses and the miscarriage rate after the 1st euploid embryo transfer (OR = 1.21; 95% CI 1.00-1.46; *P* = 0.04).

**Table 3:** Logistic regression model to evaluate associations between the clinical outcomes of the 1st FET and previous IVF failed attempts

	[n (%)]	aOR	95% CI	*P* value
Livebirth	115 (41.1)	0.76	0.62–0.94	0.01
Pregnancy	149 (53.0)	0.59	0.32–1.07	0.08
Miscarriage	32 (21.8)	2.50	0.96–6.62	0.06

aOR adjusted odds ratio; CI confidence interval

Adjusted for age and the number of embryos transferred

## Discussion

The experience of recurrent implantation failure represents a heavy burden for couples seeking IVF success. The transfer of euploid blastocysts to improve reproductive outcomes has minimized the risks of recurrent implantation failure in women undergoing IVF treatment [13]. Our study aimed at generating valuable data to test this hypothesis in a university-based IVF practice to support patient counseling and inform practice-based recommendations. Our findings showed that the LBR after the first vitrified-warmed euploid blastocyst transfer demonstrated a decreasing trend based on the number of previous IVF failures, which became significant in women with a history of three or more failed attempts. The LBR of successive euploid embryo transfers also dropped from 37.1 to 25.0% after the 3rd FET cycle (*P* = 0.02). We also showed that at least 20% of our patient population failed to achieve sustained implantation after the transfer of up to three euploid blastocysts. These findings are in stark contrast with the study of Pirtea et al. [13] which showed that clinical outcomes of euploid embryos do not vary significantly following successive transfer cycles and that < 5% of women fail to achieve implantation with three euploid embryos transferred. The investigators questioned the rare existence of RIF and entertained a statistical certainty based on random variation. In contrast, our findings indicate that the viability of an embryo is dependent on other factors that go beyond its euploidy status. It is reasonable to believe that much of an embryo’s success is determined by patients’ characteristics, endometrial variables, and parameters associated with embryo handling, such as the culture environment of the laboratory, the skills of the embryologist, and the expertise of the physician. In the present study, statistically significant interactions were detected between patient’s characteristics and the reproductive outcomes of the 1st FET. We found that the number of previous IVF failed attempts was associated with a lower livebirth rate (OR = 0.76; 95% CI 0.62–0.94; *P* = 0.01), and that the number of previous pregnancy losses was associated with a higher miscarriage rate (OR = 1.21; 95% CI 1.00-1.46; *P* = 0.04). Furthermore, discrepancies between the findings of different studies may suggest that the viability of a euploid embryo is highly dependent on the success of individual IVF practices. Findings published by some centers therefore may not always qualify for generalization. The ∼20% implantation failure demonstrated after three transferred embryos, supports the traditional definition of RIF and suggests that IVF success is a sum of factors that characterize individual practices adding up to the embryo euploid status. The adoption of a universal definition for RIF may hence be misleading and could result in the potential loss of the precious opportunity to troubleshoot, finetune and adjust the success rate. We, therefore, call on adopting a definition for RIF that reflects the unique characteristics of the population served and the clinical performance of the individual practice. A context-based definition may better reproduce the sum of events that characterize an individual practice. It should be noted that while mathematical modeling has been proposed to create individualized practice-based predictive pregnancy outcome models [19–21], these simple formulas often fail to account for the temporal attrition associated with multiple unsuccessful attempts [22].

While a strong inverse association was established between the euploidy status of embryos and maternal age, an association with the number of failed IVF attempts was not established. Our study failed to show a difference in the mean euploidy rate per cohort of biopsied blastocysts according to the number of previously failed IVF cycles (0/1–2/≥3) within different ranges of maternal age at oocyte retrieval. By accounting for the contribution of the euploid status of embryos in PGT-A cycles, it becomes possible to better understand the relative influence of other potential variables in the process of implantation. Our findings are congruent with those of others, which suggest a significant role for factors beyond the euploidy state in the pathogenesis of recurrent implantation failure [16, 18, 23]. We further demonstrated that the live birth rate of the first transferred euploid blastocyst was sensitive to the number of previous IVF failures, dropping significantly from 45.3 to 27.6% in women with a history of three previous failed attempts. These findings agree with those of others who reported a lower LBR in patients with RIF [23]. The significant drop in the live birth rates of successive vitrified-warmed euploid blastocysts (37.1% vs. 25%; *P* = 0.02) also supports the presence of an inherent pathology in women with repeated IVF failed attempts and may uncover the etiological significance of a potential endometrial contributor. Precision diagnostic tools recently revealed some molecular characteristics of endometrial dysfunction in women with RIF [24, 25]. Endometrial tissues derived from this patient population have shown major disruptions to signaling pathways and gene functions involved in endometrial receptivity and embryo implantation [24, 26, 27].

Considering the predominantly idiopathic nature of RIF, the most challenging question is about the timing and nature of any investigative work-up [3–5]. Patient investigations seeking possible causes of RIF are costly and often non-yielding, invariably resulting in overdiagnosis and overtreatment. The initiation of a work-up should therefore be weighed against the likelihood of finding a causative etiology and the benefits of the proposed interventions. Since the euploid status of embryos was shown to be the major determinant of embryo success, the transfer of euploid embryos was proposed as a means of closing the gap between non-identified contributing factors and the law of probability. PGT-A was therefore proposed as an acceptable approach to manage RIF [13, 17, 18]. Our findings showed that the LBR after the first vitrified-warmed euploid blastocyst transfer manifested a decreasing trend based on the number of previous IVF failures, which was significant in women with a history of three or more failed attempts. The LBR of successive euploid embryo transfers also dropped significantly from 37.1 to 25% after the 3rd FET cycle (*P* = 0.02). These findings confirm the significant contribution of factors, other than the chromosomal profile of an embryo, to the process of implantation. In the present study, significantly more good-quality embryos were replaced in the 1st FET compared to the 3rd FET (94.8% vs. 76.5%; *P* = 0.01). Considering the likelihood that embryos transferred on the 1st FET attempt undergo a strict selection process based on morphokinetics criteria, it is hence reasonable to estimate that the difference in outcomes between successive FET cycles may be in part accounted for by embryo selection criteria. Embryonic factors such as mitochondrial DNA constitution [28] and embryo developmental kinetics [29] also represent important determinants of the implantation process. With the introduction of new ‘omics’ technologies, a deeper dive into the endometrial molecular signatures could also prove to be very revealing about the role of the endometrium in this context. This may explain the reasons why some studies have failed to confirm a role for PGT-A in the management of recurrent implantation failure [11, 23]. While acknowledging the relative differential contribution of potential etiologies, it is reasonable to propose PGT-A as a pragmatic approach for a select group of RIF women, namely those who present at a relatively advanced age with good ovarian reserve. In this case, chromosomal screening could offer the opportunity to reduce the number of transfers with non-viable embryos, with the clear benefit of shortening the time interval to pregnancy.

### Limitations

We acknowledge that our findings are limited by the observational nature of the study design, which is associated with a significant risk of bias hampering the strength of the final conclusions. The limited size of the patient cohort and the fact that all successive cryo-warmed transfers were derived from the same oocyte retrieval attempt, may further compromise the merits of the study. The decline of the reproductive performance of successive blastocyst transfers is believed to be the result of the embryo selection process favoring the first embryo transfer. Furthermore, the small number of observations in the 3rd FET cycles could undermine the strength of the findings. To circumvent the risk of bias introduced by the failure of some patients to replace their remaining embryos following their first failed attempt, these were assigned the same outcomes associated with each successive transfer cycle.

## Conclusions

The implantation potential of vitrified-warmed euploid blastocysts collected from the same oocyte retrieval cycle declines significantly after each consecutive embryo transfer. This fact, beside the inverse association between the clinical outcomes of the first euploid embryo transfer and the number of previous IVF failures suggests the existence of a clinical entity characterized by the failure of implantation despite repeated embryo transfers. Since the uterine transfer of euploid embryos does not seem to effectively address this condition, it is needed to look into other etiologic causes beyond the chromosomal status of embryos. At least one in five women in the study population failed to achieve sustained implantation after the transfer of up to three euploid embryos. Further studies are required to gain more insight into the causes of recurrent implantation failure.

## Data Availability

No datasets were generated or analysed during the current study.

## Abbreviations

RIF: Recurrent implantation failure
ET: Embryo transfer
IVF: In vitro fertilization
PGT-A: Pre-implantation genetic testing for aneuploidy
TE: Trophectoderm
FET: Frozen embryo transfer
IRB: Institutional review board IRB
COS: Controlled ovarian stimulation
ICSI: Intracytoplasmic sperm injection
GnRH: Gonadotropin releasing hormone
SD: Standard deviation
CI: Confidence intervals
SIR: Sustained implantation rate
LBR: Livebirth rate
m-ER: Mean euploidy rate
hCG: Human chorionic gonadotropin
